# On the Edge of Psychopathology: Strong Relations Between Reversed Self-compassion and Symptoms of Anxiety and Depression in Young People

**DOI:** 10.1007/s10567-024-00471-w

**Published:** 2024-03-12

**Authors:** Peter Muris, Iván Fernández-Martínez, Henry Otgaar

**Affiliations:** 1 Department of Clinical Psychological Science, Faculty of Psychology and Neuroscience, Maastricht University, P.O. Box 616, 6200 MD Maastricht, The Netherlands; 2 Stellenbosch University, Stellenbosch, South Africa; 3 Miguel Hernández University, Elche, Spain; 4 Catholic University Leuven, Leuven, Belgium

**Keywords:** Self-compassion, Compassionate and uncompassionate self-responding, Anxiety and depression, Adolescents

## Abstract

Self-compassion is assumed to have a protective role in the etiology of emotional problems in adolescents. This assumption is primarily based on correlational data revealing negative correlations between the total score on the Self-Compassion Scale (SCS) and symptom measures of anxiety and depression. Recently, however, the SCS has been criticized because this scale not only consists of items measuring compassionate self-responding (i.e., self-kindness, common humanity, and mindfulness), but also includes ‘reversed’ items measuring uncompassionate self-responding (i.e., self-criticism, isolation, and overidentification), which would undermine the validity of the scale as an index of a protective construct. The present article used two methods to demonstrate that compassionate (positive) and uncompassionate (negative) self-responding have differential effects on emotional problems in youths. In the first part, a meta-analysis based on 16 relevant studies demonstrated a modest protective effect of positive self-compassion on anxiety/depression and a large (and significantly stronger) vulnerability effect of negative self-compassion on such emotional symptoms. In the second part, network analyses were conducted on three previously collected data sets and these analyses again showed that negative self-compassion is more closely connected to young people’s symptoms of anxiety and depression than positive self-compassion. It is argued that the observed differential effects should not be discarded as a subversive fallacy, but rather offer an opportunity for studying the role of self-compassion in adolescents’ emotional psychopathology in a more sophisticated way, taking into account both protection and vulnerability.

## Introduction

Stress is an inevitable experience in adolescents’ life: In the process of meeting expectancies at school, establishing and maintaining positive peer relations, and developing one’s own identity, many young people are faced with feelings of failure and inadequacy every now and then (Slavich et al., 2019). Although such strains have been demonstrated to be associated with the development of mental health problems including anxiety and depression (Pollmann et al., 2022), it is also true that the way an individual copes with adversity is decisive for the eventual (positive or negative) outcome (Compass et al., 1993). During the last decades, a vast amount of research has been devoted to the positive psychology construct of self-compassion, which briefly entails the perseveration of a warm, supportive, and calm attitude toward oneself when facing difficult times (Neff, 2023). More precisely, a self-compassionate attitude consists of three components: (1) self-kindness, which refers to the tendency to be caring and understanding with oneself when confronted with personal failures and problems, (2) common humanity, which is concerned with the recognition that one’s failure and difficulties are a normal part of life and that all people face such hardship every now and then, and (3) mindfulness, which pertains to the ability to face difficulties and associated negative feelings in a balanced way, being still able to pay attention to the positive things in one’s life (Neff, 2003a). There is abundant evidence indicating that adult individuals characterized by high levels (versus low levels) of self-compassion are less prone to develop emotional psychopathology (MacBeth & Gumley, 2012), and this has also been repeatedly shown in adolescent samples (Marsh et al., 2018).

While acknowledging the potential of self-compassion as a protective construct for adolescent mental health, critique has been raised regarding the way this variable has been measured in psychological research (e.g., Brenner et al., 2018; Lopez et al., 2015; Muris & Otgaar, 2020; Pfattheicher et al., 2017). Specifically, the widely used Self-Compassion Scale (SCS; Neff, 2003b), its abbreviated equivalent (the SCS-Short Form or SCS-SF; Raes et al., 2011), as well as the more recently developed age-downward version (the SCS for Youth or SCS-Y; Neff et al., 2021) all contain a considerably number of items (i.e., SCS: 13 out of 26, SCS-SF: 6 out of 12; SCS-Y: 8 out of 17) that do not assess the positive components of self-kindness, common humanity, and mindfulness, but rather measure their exact counterparts, namely self-judgment, isolation, and overidentification. In general, this is not a problem—as it may be interesting and relevant to study the differential effects of compassionate as well as uncompassionate self-responding on people’s adjustment to adversity. The problem lies in the fact that the vast majority of researchers do not use the separate components of the scale but rather rely on the scale’s total score in which the reversed uncompassionate self-responding items are included. In our opinion, this is a doubtful practice that poses serious threat to the validity of the measure as an index of a fully protective construct (Muris & Otgaar, 2020, 2022).

It can be argued that the negative components of the SCS, SCS-SF, and SCS-Y tap a number of ‘toxic mechanisms’ (Muris, 2016; p. 1464) or even ‘pervasive features of mental health problems’ (Muris et al., 2016; p. 789) that are difficult to reconcile with the true, protective nature of self-compassion. To illustrate this point, Table 1 displays Neff’s (2003a) definitions of self-judgment, isolation, and overidentification (and sample items of the original SCS) as well as a number of associated psychological constructs. As can be seen, self-judgment bears similarity to self-blame, self-criticism, self-punishment, negative self-image, and the psychodynamic defense mechanism of ‘turning against the self’; isolation relates to loneliness, an insecure attachment status, and social avoidance and withdrawal; while overidentification shares features with repetitive negative thinking (i.e., worry and rumination), self-absorption, psychopathology-related cognitive biases, and emotional dysregulation. Each of these constructs refers to a feature or process that plays a prominent role in a variety of psychopathological conditions, but especially in anxiety disorders and depression. It is thus logical to expect that the components of self-judgment, isolation, and overidentification will be positively related to symptoms of these disorders, and this may contain the hazard that when including the reversed negative components in the total self-compassion score, its relations with emotional psychopathology will become inflated. The latter will be particularly true when correlations between the negative self-compassion components and anxiety/depression symptoms are stronger than those between positive self-compassion components and this type of emotional symptoms.

**Table 1:** Three negative components included in the SCS (and its shortened and age-downward variants), reflecting ways of *un*compassionate self-responding in people who experience failure and personal shortcomings, and a number of related ‘pathogenic’ psychological constructs

SCS Negative components (sample item)	Related constructs
Self-judgment	Self-blame
A harsh and critical attitude toward oneself (‘I am disapproving and judgmental about my flaws and inadequacies’)	Attributing the occurrence of a negative event to one’s own actions or character (Jannati et al., 2020)
	Self-criticism
	Constant and harsh self-scrunity and negative self-evaluation (Zuroff et al., 1999)
	Self-punishment
	A deliberate attempt to harm yourself physically or psychologically (Nelissen & Zeelenberg, 2009)
	Negative self-image
	A disproportionate focus on one’s faults and weaknesses (Schreiber & Steil, 2013)
	Turning against the self
	A special form of displacement, where the person becomes his own target at which anger and aggression is directed (Campos et al., 2011)
Isolation	Loneliness
Feeling disconnected from others when suffering (‘When I think about my inadequacies, it tends to make me feel more separate and cut off from the rest of the world’)	A negative emotional feeling indicating an unmet need for social connection (Cacioppo et al., 2006)
	Insecure attachment
	Due to early childhood experiences, relationships with other people lack a secure base and contain elements of distress, anxiety, and avoidance (Mikulincer & Shaver, 2012)
	Social avoidance/withdrawal
	Removing oneself from opportunities to connect with others, because of anxiety, fear, shame, vulnerability, potential rejection etc. (Teo et al., 2015)
Overidentification	Rumination/worry
Becoming absorbed by negative thoughts and feelings (‘When I am feeling down, I tend to obsess and fixate on everything that is wrong’)	Two forms of persistent and repetitive thinking about sad events that have happened in the past or frightening events that might take place in the future (Ehring & Watkins, 2008)
	Self-absorption
	An excessive, sustained, and rigid focus on the self; a preoccupation with one’s own feelings and situation (Ingram, 1990)
	Psychopathology-related cognitive biases
	Systematic errors in information processing: negative information is registered more readily and people also tend to dwell more on negative events (Mineka & Sutton, 1992)
	Emotional dysregulation
	Displaying intense emotional reactions that are out of proportion to the circumstances (Sheppes et al., 2015)

*Note*. SCS = Self-Compassion Scale

A previous meta-analysis by Muris and Petrocchi (2017)—mainly including studies (*k* = 18) that relied on adult samples—indeed demonstrated that this appears to be the case. More specifically, the pooled effect sizes for the relations with symptoms of psychopathology (mostly internalizing but also externalizing and psychotic problems) were significantly larger for the negative components of self-compassion (self-judgment: |.47|, isolation: |.50|, overidentification: |.48|, and total negative: |.48|) than for the positive components of self-compassion (self-kindness: |− 0.34|, common humanity: |− 0.27|, mindfulness: |− 0.33|, and total positive: |− 0.31|). Hence, when the reversed negative components are included in a self-compassion total score, relations with indices of psychopathology will be inflated. This implies that the commonly observed and quite robust negative relationship between self-compassion and psychopathology (MacBeth & Gumley, 2012; Marsh et al., 2018), which is suggesting a clear protective effect, can be largely explained by reversely scored vulnerability.

In our own empirical work that is primarily focused on mental health problems in youths, we have also occasionally noted that the negative components of the construct were more substantially linked to psychological problems than the positive components (e.g., Muris et al., 2021), which suggests that the inflation effect when using the total score of the SCS (or SCS-SF/SCS-Y) may also occur in younger populations. However, a systematic analysis of the literature on the possible differential links between the positive and negative self-compassion components and psychopathological symptoms in adolescents is currently lacking. With this in mind, the first part of the present article is devoted to a meta-analysis of studies that examined the relationship between self-compassion and psychopathology in youth populations, while taking into account the separate effects of the negative and positive components of this self-related construct. In terms of psychopathology, the meta-analysis was restricted to anxiety and depression, because these two types of emotional problems have been shown to be prominent reactions to adverse and stressful life experiences, especially in adolescents (e.g., Anyan & Hjemdal, 2016; Oldehinkel & Bouma, 2011; Romeo, 2013), and are thought to be most clearly associated with compassionate and uncompassionate self-responding tendencies (e.g., Neff, 2023).

Due to the primary focus on the total score of the SCS or equivalent scales (Muris & Otgaar, 2022), studies on the relative contributions of the separate or combined positive and negative self-compassion components to symptoms of anxiety and/or depression are quite sparse, and this is certainly true when considering research conducted in young people. Notable exceptions concern studies that have employed regression analysis (e.g., Liu et al., 2023; Muris et al., 2018, 2022; Stolow et al., 2016; Tali et al., 2023), which refers to a statistical method that can be used to study the unique relations between a number of independent variables (i.e., various self-compassion components) and a single dependent variable (i.e., anxiety or depression; see Field, 2009). Network analysis might provide a valuable alternative way of looking at such correlational data (Hevey, 2018). This method does not make a distinction between independent and dependent variables, but evaluates the relationships (strength and direction: positive versus negative) among constructs included in a model, while controlling for all other variables (Epskamp et al., 2018). Applying this to research on the links between negative and positive self-compassion components and symptoms of anxiety and depression, such an approach would account better for the complexity of a psychopathology model, involving the relations between protective (i.e., positive self-compassion) and vulnerability (negative self-compassion) factors as well as various types of emotional symptoms, which have also been demonstrated to covary considerably with each other (e.g., Brady & Kendall, 1992).

Interestingly, Deniz et al. (2022) recently adopted a network analysis approach to explore the relations among various self-compassion components as measured by the SCS-Y and depression in a sample of 11- to 15-year-old Turkish youths. The pattern of the correlations showed that the self-compassion components clustered in two separate categories, with a single substantial (expected) negative correlation between self-kindness and self-judgment. Most importantly, it were mainly the negative components that appeared to be related to depression symptoms, which provides further support that in particular uncompassionate self-responding tendencies are relevant in the study of this type of emotional problem. Meanwhile it should be acknowledged that the network model tested by Deniz et al. was not a pure psychopathology model as it also included a number of positive constructs, namely happiness and resilience, which may have resulted in diminution of some relations. For example, the expected negative links between the positive self-compassion components and depression might not have emerged because happiness accounted for most of the ‘protective’ variance in the model. With this in mind, the second part of the present article adopts a network analysis approach to study the (unique) relations among the positive and negative components of self-compassion and adolescents’ mental health, thereby focusing on the emotional psychopathology constructs of anxiety and depression (Kircanski et al., 2017).

To recap, the present article consists of two parts: a meta-analysis and a network analysis. In the meta-analytic part, the extant literature was reviewed to select studies that examined the correlations between the positive and negative components of self-compassion—as measured with the SCS or its variants—and symptoms of anxiety and/or depression in adolescents. In line with the results of our previous meta-analysis that mainly included studies with adults samples (Muris & Petrocchi, 2017), we expected to document negative relations between the (combined and separate) positive self-compassion components and symptom measures of anxiety and depression in young people (indicating a ‘protective’ effect), while we anticipated positive relations between the negative self-compassion components and such emotional symptom measures (reflecting a ‘vulnerability’ effect). Moreover, we hypothesized that relations between negative components of self-compassion and anxiety/depression would be stronger than those between positive components and emotional problems, which would imply that the earlier described ‘inflation effect’ (Muris & Otgaar, 2020, 2022)—when using the reversed negative components for computing a SCS total score—is also present when conducting research on self-compassion in youth populations.

In the network analysis part, we analyzed three existing data sets (Muris et al., 2018, 2021, 2022)—each relying on a different version of the SCS (i.e., the full-length SCS, the SCS-SF, and the SCS-Y)—to take a detailed look at the unique intercorrelations between the positive and negative self-compassion components and symptoms of anxiety and depression in young people. Here, we anticipated a strong link between anxiety and depression symptoms (Brady & Kendall, 1992), with both types of emotional symptoms showing stronger associations with the negative components than with the positive components of the self-compassion construct.

## Part 1: Meta-Analysis

### Method

On August 31, 2023, a literature search was conducted in Web of Science with [self-compassion in title] AND [child* or ado* or you* in topic] AND [anx* or depress* in topic] as the search terms. The searching period was 2003 (i.e., the year that the construct of self-compassion was first described in the scientific literature) to 2023. For all detected articles, the first author carefully checked whether: (1) the article described an empirical study; (2) the study included a standardized assessment of self-compassion by means of the SCS or its variants; (3) the population of the study consisted of participants in the adolescent age (i.e., between 12 and 18 years; studies with a somewhat broader age range were accepted in case the average age of the participants fell within this range); (4) the study included a standardized measurement of anxiety and/or depression symptoms (in case of anxiety, various constructs such as fear, worry, trait anxiety, and social anxiety were accepted); and (5) the article reported the correlations between self-compassion and anxiety and/or depression symptoms, while making a distinction between the positive and negative aspects of the self-compassion construct as per Neff’s (2003a) definition.

From all the selected articles, the following data were extracted: study and sample characteristics (i.e., author(s) plus year of publication, sample size, gender distribution, age range and mean age, country in which study was conducted), the version of the SCS that was used and the measure(s) of anxiety and/or depression, and the zero-order correlations between the positive and negative self-compassion indicators and anxiety and/or depression. Next, Wilson’s (2010) online meta-analysis effect size calculator was used to calculate Fisher’s *z*-transformed correlations (*r*) and accompanying 95% confidence interval (CI) as an effect size indicator for the correlations between (a) the separate positive and negative self-compassion components (as indexed by subscales of the SCS or its variants) and indices of anxiety and/or depression; and/or (b) the combined positive and negative self-compassion components and these symptom measures that were reported in each individual study. Fisher’s *z*-transformed correlations and CIs were eventually averaged across all studies. We expected to document negative effect sizes (i.e., a protective effect) for the relations between (separate and combined) positive self-compassion components and indices of anxiety and depression, whereas we anticipated positive effect sizes (i.e., a vulnerability effect) for the relations between (separate and combined) negative self-compassion components and measures of these emotional problems.

The meta-analysis was not preregistered. However, we closely followed the ‘Preferred Reporting Items for Systematic reviews and Meta-Analyses’ (PRISMA; Page et al., 2021) guidelines. Also, a post hoc evaluation using the ‘A MeaSurement Tool to Assess systematic Reviews’ (AMSTAR) instrument (Shea et al., 2007) revealed that our meta-analysis fulfilled most critical criteria and hence was of good quality. More specifically, the research question was focused, the literature search was comprehensive, the inclusion criteria were clear (i.e., an initial check by the third author of the 16 included and 16 randomly selected excluded papers revealed a percentage of agreement of 96.9%, with a kappa value of 0.94; a second more thorough analysis by the same person increased the agreement to 100%, kappa value = 1), the data extraction was straightforward, a well-known effect size calculator method was used for the meta-analysis, and the likelihood of publication bias was evaluated by means of funnel plots.

## Results

**Fig. 1:**
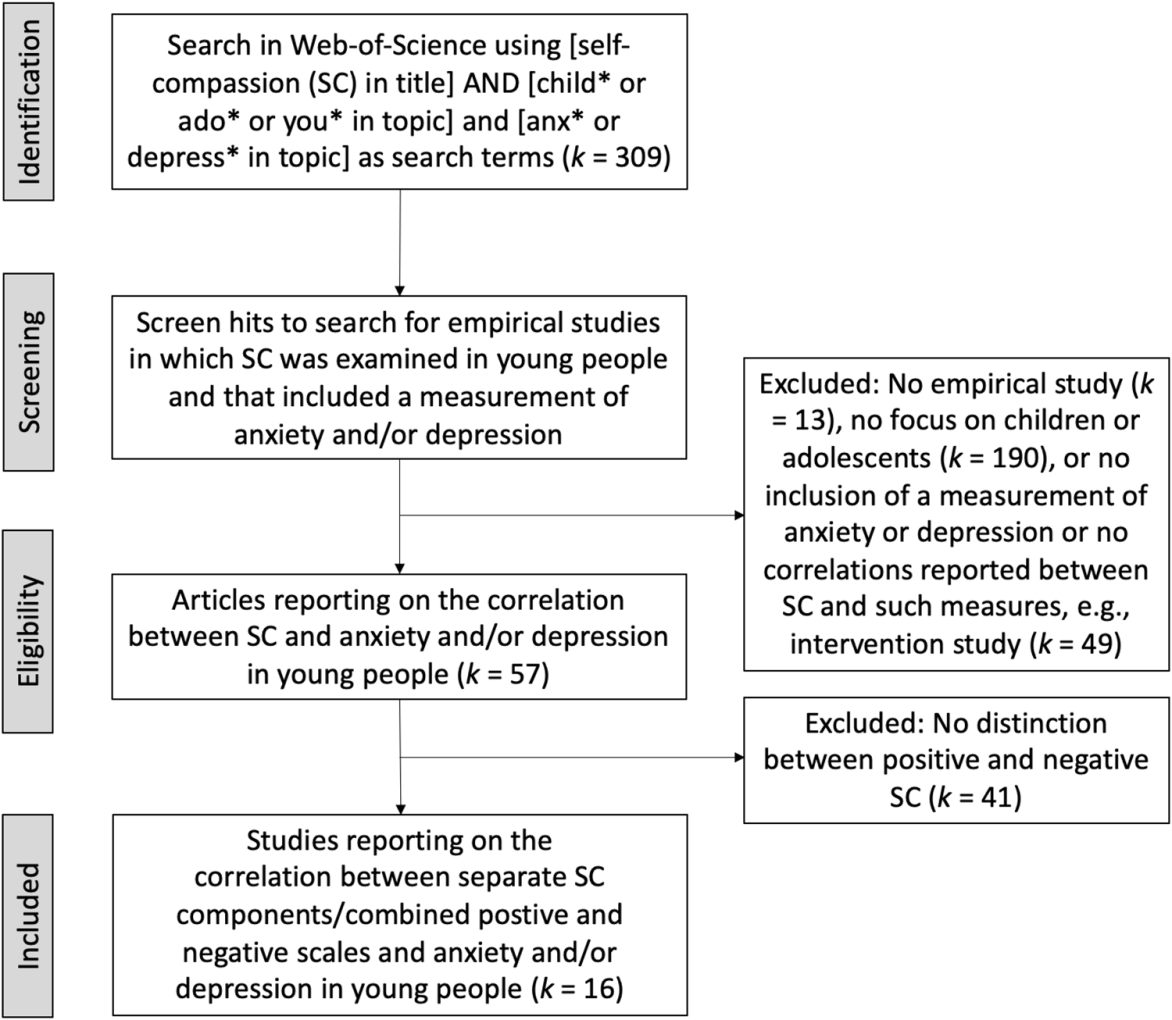
PRISMA flow diagram depicting the selection of articles that were included in the meta-analysis of the relations between positive and negative self-compassion components and anxiety/depression in young people

Figure 1 shows the PRISMA flow diagram of our literature search and the subsequent steps that led to the ultimate inclusion of studies in the meta-analysis. As can be seen, the initial search yielded 309 publications that were all subjected to a careful inspection. A total of two-hundred-and-fifty-two articles were discarded because they did not report on an *empirical* study on self-compassion (*k* = 13), were focused on adults (e.g., parents) rather than on adolescents (*k* = 190), did not include a measurement of anxiety and/or depression, or were not correlational in nature and hence did not report correlations between self-compassion and such emotional symptoms (*k* = 49). The 57 studies that were left all reported on the correlation(s) between self-compassion (as measured with the SCS or one of its variants) and anxiety and/or depression in young people. However, more than two-thirds of these investigations (*k* = 41) merely reported on the self-compassion total score and hence did not make a distinction between the positive and negative self-compassion in relation to these emotional problems, which was the key focus of our meta-analytic endeavor.

Thus, eventually, 16 studies were identified as relevant for our meta-analysis. A summary description of these studies are given in Table 2. The total number of young people included in these studies was 10,379 (5050 males, 5325 females, and 4 unidentified), of which the vast majority was aged between 12 and 18 years. Most studies relied on non-clinical participants who were recruited from the community via the school system or the internet (*k* = 13), but there were three investigations that made use of clinically referred (Barry et al., 2015; Tali et al., 2023) or at risk (Liu et al., 2023) youngsters. Self-compassion was mostly assessed by means of Neff’s (2003b) original SCS (*k* = 11); in other studies, the shortened (SCS-SF; *k* = 3) or age-downward (SCS-Y; *k* = 3) were (also) employed. From 10 studies, scores for the separate positive (i.e., self-kindness, common humanity, and mindfulness) and negative (i.e., self-judgment, isolation, and overidentification) as well as combined (i.e., total positive and total negative) self-compassion components could be derived, whereas the other investigations (*k* = 6) only reported scores for combined positive and negative self-compassion components. A variety of measures were used to measure symptoms of anxiety and depression in the young participants: eight studies assessed both types of symptoms, five studies only measured symptoms of depression, whereas three studies only focused on anxiety.

**Table 2:** Summary description of the studies that were included in the meta-analysis of the relations between positive and negative self-compassion components and anxiety/depression in young people

Study	Sample characteristics	Self-compassion measure	Anxiety and/or depression measure
Barry et al. (2015)	251 clinically referred adolescents (all males), 16–18 years, *M* age = 16.78 years, USA	SCS: separate positive and negative components as well as combined positive and negative scales	Depression and Fear/worry subscales of the Personality Inventory for Youth (PIY)
Cunha et al. (2016)	3165 non-clinical adolescents (1461 males and 1704 females), 12–19 years, *M* age = 15.49 years, Portugal	SCS: separate positive and negative components as well as combined positive and negative scales	Depression and anxiety subscales of the Depression Anxiety and Stress Scales (DASS-21)
Einstein et al. (2023)	951 non-clinical adolescents (509 males and 442 females), 12–16 years, *M* age = 13.69 years, Australia	SCS-SF: combined positive and negative scales	Spence Children’s Anxiety Scale
Ferrari et al., (2023a, 2023b)	929 non-clinical adolescents (495 males and 434 females), 12–16 years, *M* age = 13.70 years, Australia	SCS-SF: separate positive and negative components as well as combined positive and negative scales	Spence Children’s Anxiety Scale; Short Mood and Feelings Questionnaire
Gill et al. (2018)	316 non-clinical adolescents (170 males and 146 females), 14–18 years, *M* age = 14.77 years, UK	SCS: separate positive and negative components as well as combined positive and negative scales	Social Phobia Inventory; Fear of Negative Evaluation subscale of the Social Anxiety Scale for Adolescents
Gruber et al. (2023)	255 non-clinical children and adolescents (104 males and 151 females), 10–19 years, *M* age = 14.90 years, Germany	SCS: separate positive and negative components as well as combined positive and negative scales	Anxiety and depression subscales of the Youth Self-Report
Henje et al. (2024)	316 non-clinical adolescents (103 males and 213 females), 15–20 years, *M* age = 17.07 years, Sweden	SCS: separate positive and negative components as well as combined positive and negative scales	Depression scale of the Beck Youth Inventories; Revised Child Anxiety and Depression Scale
Lahtinen et al. (2020)	2383 non-clinical adolescents (1134 males and 1249 females), 16–18 years, Finland	SCS: combined positive and negative scales	Revised Beck Depression Inventory
Liu et al. (2023)	450 adolescents exposed to an earthquake (206 males and 244 females), *M* age = 13.72 years, China	SCS: combined positive and negative scales	Center for Epidemiological Studies Depression Scale
Muris et al. (2018)	130 non-clinical adolescents (44 males and 86 females), 15–19 years, *M* age = 16.68 years, the Netherlands	SCS: separate positive and negative components as well as combined positive and negative scales	Trait anxiety version of the State-Trait Anxiety Inventory for Children; Children’s Depression inventory
Muris et al. (2021)	158 non-clinical adolescents (69 males, 85 females, and 4 unknown), 11–17 years, *M* age = 13.54 years, the Netherlands	SCS and SCS-SF: combined positive and negative scales	Youth Anxiety Measure for DSM-5; Children’s Depression Inventory
Muris et al. (2022)	87 non-clinical adolescents (26 males and 61 females), 12–18 years, *M* age = 15.28 years, the Netherlands	SCS-Y: separate positive and negative components as well as combined positive and negative scales	Youth Anxiety Measure for DSM-5; Children’s Depression Inventory
Nazari et al. (2022)	532 non-clinical adolescents (270 males and 262 females), 12–15 years, *M* age = 13.57 years, Iran	SCS-Y: separate positive and negative components as well as combined positive and negative scales	Depression items of the Patient Health Questionnaire
Neff et al. (2021)	212 non-clinical adolescents (112 males and 90 females), 11–14 years, *M* age = 12.18 years, USA	SCS-Y: separate positive and negative components as well as combined positive and negative scales	Center for Epidemiological Studies Depression Scale
Stolow et al. (2016)	193 non-clinical children and adolescents (79 males and 114 females), 9–16 years, *M* age = 13.04 years, USA	SCS: combined positive and negative scales	Children’s Depression Inventory
Tali et al. (2023)	23 clinically referred adolescents with an anxiety disorder (7 males and 16 females) and 28 non-clinical controls (10 males and 18 females), 12–19 years, *M* = 15.26 years, the Netherlands	SCS: combined positive and negative scales	State-Trait Anxiety Inventory for Children

SCS = Self-Compassion Scale, SCS-SF = Self-Compassion Scale–Short Form, SCS-Y = Self-Compassion Scale for Youth

As given in Table 3, the average correlations between the combined positive self-compassion components and indices of anxiety and depression were negative: *r*’s being -0.19 and -0.25, respectively, with associated effect sizes being -0.20 and -0.26, both indicating a small ‘protective’ effect. The average correlations between the combined negative self-compassion components and measures of anxiety and depression were positive: *r*’s being 0.53 and 0.50, respectively, with associated effect sizes being 0.61 and 0.56, both reflecting a quite large ‘vulnerability’ effect. This pattern of results was also evident when looking at the results for the separate positive and negative self-compassion components: more precisely, self-kindness, common humanity, and mindfulness were all modestly, negatively linked to anxiety and depression (range of *r*’s between − 0.08 and − 0.36, with effect sizes between − 0.08 and − 0.40), whereas self-judgment, isolation, and overidentification were all substantially, positively associated with these emotional problems (range of *r*’s between 0.45 and 0.51, with effect sizes between 0.49 and 0.57). The observed relations appeared to be quite robust: Average correlations/effect sizes were all significant at *p* < 0.001, and in the majority of cases, the confidence interval associated with the effect size did not include ‘0,’ the one exception being the positive self-compassion component of common humanity in relation to anxiety symptoms (see Table 3). Note also that data showed considerable heterogeneity (all Q-statistics were significant at *p* < 0.001), which implies that findings varied considerably across studies.

**Table 3:** Results of the meta-analysis of studies on the relations between separate/combined positive and negative self-compassion (SC) components and measures of anxiety and depression in children and adolescents

	*k*	*N*	Average *r*	Effect size *r*	95% CI	*p*	Heterogeneity *Q*^†^
*Anxiety*							
Combined positive SC components	11	5403	− 0.19	− 0.20	− 0.33, − 0.07	< .001	91.06
Self-kindness	8	4243	− 0.26	− 0.27	− 0.39, − 0.16	< .001	79.83
Common humanity	8	4243	− 0.08	− 0.08	− 0.20, 0.04	< .001	32.25
Mindfulness	8	4243	− 0.19	− 0.20	− 0.32, − 0.08	< .001	24.52
Combined negative SC components	11	5403	0.53	0.61	0.48, 0.74	< .001	78.79
Self-judgment	8	4243	0.51	0.57	0.46, 0.69	< .001	142.98
Isolation	8	4243	0.47	0.51	0.39, 0.63	< .001	69.81
Overidentification	8	4243	0.51	0.56	0.44, 0.68	< .001	92.25
*Depression*							
Combined positive SC components	13	7855	− 0.25	− 0.26	− 0.38, − 0.15	< .001	129.00
Self-kindness	9	4671	− 0.36	− 0.40	− 0.51, − 0.28	< .001	174.03
Common humanity	9	4671	− 0.15	− 0.16	− 0.28, − 0.04	< .001	62.97
Mindfulness	9	4671	− 0.24	− 0.25	− 0.37, − 0.13	< .001	68.93
Combined negative SC components	13	7855	0.50	0.56	0.44, 0.67	< .001	215.72
Self-judgment	9	4671	0.49	0.55	0.43, 0.67	< .001	173.13
Isolation	9	4671	0.47	0.53	0.41, 0.65	< .001	221.17
Overidentification	9	4671	0.45	0.49	0.37, 0.61	< .001	198.11

^†^Heterogeneity was evaluated by means of the software package Jamovi (https://www.jamovi.org). The *Q*-statistic was significant for all analyses

The inspection of the funnel plots revealed that between 25 and 66.7% of the studies fell outside the 95% confidence interval, again pointing at considerable variation in effect sizes across studies (independent of the sample size and the precision of the estimated effect size). Furthermore, it is important to mention that the points in the funnel plot were located equally on the left and the right side of the overall effect, suggesting that data were not systematically biased in either direction (Egger et al., 1997). However, it should be borne in mind that a substantial number of studies on self-compassion and anxiety/depression in young people (*k* = 41) were not included in the present meta-analysis (as they did not report separate correlations for the negative and positive self-compassion components), implying that the present data only show the ‘tip of the iceberg’ concerning the relation between self-compassion and anxiety/depression in adolescents. Nevertheless, the outcomes of individual studies were generally in the same direction as the average outcomes and well in line with presumed protective and vulnerability nature of positive and negative self-compassion, giving us confidence about the validity of the observed effects.

Tests for comparing correlation coefficients (Meng et al., 1992) were conducted to evaluate differences between positive and negative components of self-compassion with regard to the strength of their relation with anxiety/depression in young people (https://www.psychometrica.de/correlation). To enable a direct comparison, average effect sizes for negative self-compassion components were reversed (as is usually done when computing a total self-compassion score for the SCS or its variants). These tests also require the intercorrelations between various positive and negative self-compassion components, which were obtained by averaging the pertinent correlations across the included studies (if reported). As shown in Table 4, the results indicated that in all cases the (combined and separate) negative self-compassion components showed a statistically significant stronger association with both types of emotional symptoms than the positive self-compassion components.

**Table 4:** Statistical comparison of the relations between positive and negative components of self-compassion (SC) and measures of anxiety/depression in young people

Anxiety	Average effect sizes (respectively)	*Z*	*p*
Combined Positive versus Negative SC components	− 0.20 versus − 0.61	27.10	< .001
Self-kindness versus Self-judgment	− 0.27 versus − 0.57	20.36	< .001
Common humanity versus Isolation	− 0.08 versus − 0.51	22.64	< .001
Mindfulness versus Overidentification	− 0.20 versus − 0.56	21.21	< .001
*Depression*			
Combined Positive versus Negative SC components	− 0.26 versus − 0.56	23.65	< .001
Self-kindness versus Self-judgment	− 0.40 versus − 0.55	11.02	< .001
Common humanity versus Isolation	− 0.16 versus − 0.53	20.93	< .001
Mindfulness versus Overidentification	− 0.25 versus − 0.49	14.55	< .001

## Discussion

The results of our meta-analysis on the relation between positive and negative self-compassion components and emotional problems in young people correspond well with the findings of our previous meta-analysis focusing on research based on adult samples (Muris & Petrocchi, 2017). That is, the combined data of the identified 16 studies again showed that the positive components of self-compassion as measured by the SCS (or its variants) were negatively associated with adolescents’ symptoms of anxiety and depression, whereas the negative components of self-compassion were positively and more substantially related to such symptoms.

When considering the magnitude of the relationships observed for the positive components, two conclusions can be drawn. First of all, the effect sizes found with depression (*r*’s between − 0.16 and − 0.40) were larger than those noted with anxiety (*r*’s between − 0.08 and − 0.27), suggesting that self-compassion has more ‘protective’ potential for the former than for the latter type of affective problems. Second, a comparison with the effect sizes obtained in the Muris and Petrocchi (2017) study revealed that the magnitude of the current study’s effect sizes for the relations involving the positive self-compassion components was considerably smaller, and this appeared especially true for common humanity (*r*’s in the present study: − 0.08 (anxiety) and − 0.16 (depression) versus *r* = − 0.27 in the Muris and Petrocchi study; *Z*’s being 8.80 and 5.29, respectively, both *p*’s < 0.001). One could argue that this was due to the fact that the present study was only concerned with symptoms of affective disorders, whereas the Muris and Petrocchi meta-analysis was based on studies that investigated a broader range of psychopathological conditions that—besides anxiety and depression—also included psychosis, eating problems, and aggression. However, this explanation is less plausible given that previous research has indicated that self-compassion is particularly relevant for affective problems such as anxiety and depression (Neff, 2003a, 2023).

Thus, it seems more appropriate to consider a developmental explanation for this finding. Given that young people are still in the process of forming a stable self and identity, it may well be that the rather complex construct of self-compassion has not fully crystallized within adolescents. Indeed, qualitative data have shown that although self-compassion in young people shares elements with the construct as manifested in adult individuals, there might also be idiosyncratic features that are tied to the developmental stage of adolescence (Klingle & Van Vliet, 2017). For example, egocentrism (including an extreme focus on the self when experiencing personal struggles) is more strongly present at an adolescent than adult age (Frankenberger, 2000), and this might explain why a sense of common humanity has not fully emerged and hence is less relevant when studying regulation of emotional symptoms in younger people. A similar line of reasoning was advanced by Seekis et al. (2022) who noted that late adolescents—when dealing with body image distress—barely mention the concept of common humanity as an affect regulation strategy.

With regard to the magnitude of the effect sizes as obtained for the negative self-compassion components, somewhat stronger associations with symptom measures were found in the current study as compared to Muris and Petrocchi (2017) study (in the present meta-analysis, effect size *r*’s were mostly in the 0.50 to 0.60 range, with an average *r* of 0.55, while Muris and Petrocchi mainly observed *r*’s in the 0.40 to 0.50 range, with an average *r* of 0.48; *Z* = 4.27, *p* < 0.001). Again, developmental considerations may be relevant here: a self-critical attitude, feelings of loneliness, and ruminative thinking (which as noted earlier cover the negative components included in the SCS or its variants) are all prototypical phenomena occurring in the ‘troubled minds’ of adolescents and may be easily fused with symptoms of anxiety and depression during this challenging stage of life (e.g., Jose & Brown, 2008; Kopala-Sibley et al., 2015; Matthews et al., 2022).

Most importantly, the present meta-analysis clearly indicated that the vulnerability effects of the negative self-compassion components surpass the protective effects of the positive components, implying that the previously described inflation effect when using the total score of the SCS (or its variants; Muris & Otgaar, 2020, 2022) is likely to occur in studies that aim to investigate self-compassion as a cognitive resilience factor within the context emotional psychopathology in youth. The results of the above-described comparisons with Muris and Petrocchi’s (2017) meta-analysis performed on data collected in adult populations (i.e., weaker links between positive self-compassion and young people’s anxiety/depression, but stronger relations between negative self-compassion and such emotional problems) suggest that research conducted in adolescent samples may be even more prone to such a methodological artifact.

It should be acknowledged that this meta-analysis was focused on investigating the cross-sectional correlations between positive and negative aspects of self-compassion and adolescents’ emotional symptoms, and interpreting these in terms of protection and vulnerability effects. However, to formally test ‘protection’ and ‘vulnerability,’ a fruitful approach would be to study participants’ emotional (i.e., anxious and/or depressive) responses within a context of stress and adverse life events. One way to achieve this would be a moderation analysis to examine whether positive and negative self-compassion interact with stress/adversity to yield (respectively) lower or higher levels of emotional symptomatology. An example of this approach is the study by Lathinen et al. (2020)—which was included in the present meta-analysis. Their results showed that positive aspects of self-compassion acted as a buffer against depression in adolescents who experienced academic difficulties, whereas no evidence was detected indicating that negative self-compassion increased the vulnerability for depression in youth encountering this type of problem. Another possibility would be to carry out longitudinal studies in which symptom development is examined in young people who have been confronted with a negative life event. For instance, Liu et al. (2023) conducted cross-lagged analyses on longitudinal data of negative and positive self-compassion and depression (which were assessed three times during a 1-year period) in 450 adolescents who had been exposed to a traumatic natural disaster (i.e., an earthquake). Positive and negative self-compassion had independent prospective effects on depressive symptoms, indicating that both components seem ‘to play a role in adolescents’ posttraumatic psychological response’ (p. 1795). Admittedly, these types of studies investigating the additive and interactive effects of positive and negative self-compassion on young people’s adaptation to stress are sparse, but are a promising next step in research on self-compassion and (emotional) psychopathology.

A related point is concerned with the interpretation of the correlations between positive and negative self-compassion and symptoms of anxiety and depression. That is, the negative correlation between positive self-compassion and emotional symptoms could point at the presence of a protective effect (i.e., high positive self-compassion shielding against symptoms of anxiety and depression) but might also be indicative for a vulnerability effect (i.e., low positive self-compassion promoting symptoms of anxiety and depression). Likewise, the positive correlation between negative self-compassion and emotional symptoms could be interpreted as a vulnerability effect (i.e., high negative self-compassion signifying greater proneness to symptoms) but could also imply a protective effect (i.e., low negative self-compassion being associated with lower symptom levels; see Seekis et al., 2021). One way to further examine this interpretation issue could be to initiate more research in clinical populations. For example, studies comparing SCS (or it variants) scores of clinically referred and non-clinical individuals could yield important information on the relative levels of positive and negative self-compassion in (young) people with affective disorders. An interesting study in this regard was conducted by Tali et al. (2023) who noted that adolescents with an anxiety disorder (*n* = 23) displayed significantly lower total self-compassion scores on the short form SCS than their non-clinical counterparts (*n* = 28). Further analysis revealed that this finding was not due to the difference in positive self-compassion scores, but was mainly explained by the difference in negative self-compassion: Adolescents with an anxiety disorder displayed higher levels of negative self-compassion than the adolescents in the non-clinical control group. Admittedly, the anxious sample in the Tali et al. study was fairly small and most of these adolescents had social anxiety disorder as primary diagnosis (47.8%), so it remains to be seen whether similar findings can be found in young persons with other affective disorders (e.g., generalized anxiety disorder or depression), but at least the results suggest that young people with anxiety problems exhibit elevated levels of negative self-compassion, which could be an important target for a psychological intervention (Egan et al., 2022).

## Part 2: Network Analysis

### Method

Network analysis is a statistical approach that can be used to study the relations among psychopathological symptoms/conditions and protective/risk factors that are thought to play a role in the etiology of these phenomena (Robinaugh et al., 2020; see Barcaccia et al., 2020 and Wolters et al., 2023 for other examples of such studies). Network models consist of *nodes*, which represent the variables under investigation (in the present study: anxiety, depression, the three positive self-compassion components of self-kindness, common humanity, and mindfulness, and the three negative self-compassion components of self-judgment, isolation, and overidentification), and *edges*, which refer to the relations among the nodes. Edges are in fact correlations between two nodes that are controlled for all other variables included in the model (i.e., partial correlations; Epskamp et al., 2018).

In order to estimate the network models, we specified a Gaussian graphical model (GGM), by using a graphical lasso (glasso) algorithm with the extended Bayesian information criterion (EBIC) model. This was done by means of the R package Bootnet with the function ‘estimateNetwork.’ The default EBICglasso was specified and the tuning parameter was set to 0.5 (Epskamp & Fried, 2018). Additionally, visualization was done using the R package Qgraph, with circles representing the nodes and lines between circles representing the edges. Blue edges indicate positive relationships between nodes, whereas red edges refer to negative relationships. The thickness of the line indicates the strength of the (positive or negative) relationship, with spurious edges being set to zero and eliminated from the model.

The centrality strength index was used to identify important nodes in the network model. Briefly, this index shows how strongly each node is directly connected to other nodes in the network, which is computed by summing all absolute weights of edges connected to that pertinent node. These values are standardized and higher values are indicative for greater centrality in the network (Opsahl et al., 2010).

Three of our own data sets were used for estimating the network model to better understand the (unique) relations among positive and negative self-compassion components and symptoms of anxiety and depression in adolescents. Muris et al. (2018) assessed self-compassion by means of the original SCS (Neff, 2003b), anxiety with Spielberger Trait Anxiety Inventory for young people (STAI; Spielberger, 1973), and depression with the Child Depression Inventory (CDI; Kovacs, 1985) in 130 adolescents aged 15 to 19 years. Muris et al. (2021) and Muris et al. (2022) used the SCS-SF (Raes et al., 2011) and the SCS-Y (Neff et al., 2021) to measure self-compassion in youth aged 12 to 17/18 years (*N*’s being 106 and 87, respectively), whereas the Youth Anxiety Measure for DSM-5 (YAM; Muris et al., 2017) and the CDI were employed in both of these studies as indices of anxiety and depression (for further details of the samples, see the pertinent articles).

## Results

**Fig. 2:**
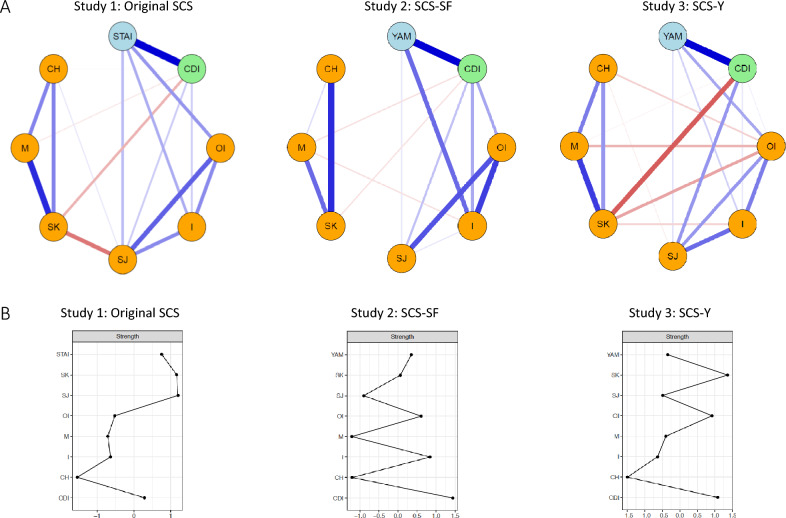
Results of the network analysis exploring the relations among positive and negative self-compassion components and emotional psychopathology in three data sets, relying on different variants of the SCS. A. Estimated network structures with orange nodes for self-compassion components, light blue nodes for anxiety, green nodes for depression, blue edges for positive relationships, and red edges for negative relationships. B. Strength centrality for various nodes in the three models; higher values indicate that a node is more strongly connected to other nodes. *Note*. Sample sizes were *N* = 130 for Study 1, *N* = 106 for Study 2, and *N* = 87 for Study 3. SCS = Self-Compassion Scale, SCS-SF = Self-Compassion Scale–Short Form, SCS-Y = Self-Compassion Scale for Youth. STAI = Spielberger Trait Anxiety Inventory, YAM = Youth Anxiety Measure, CDI = Children’s Depression Inventory, CH = common humanity, M = Mindfulness, SK = self-kindness, SJ = self-judgment, I = isolation, OI = overidentification

Figure 2 shows the networks that were estimated using the data collected in the three studies. A number of conclusions can be drawn from Panel A of this figure. First, in all three models, there was a strong positive relationship between young people’s anxiety and depression symptoms. Second, edges among positive self-compassion components and edges among negative self-compassion components were mostly positive and robust; exceptions were noted for the model of Study 2, which relied on the SCS-SF, revealing rather weak edges between common humanity and mindfulness and between self-judgment and isolation. Third, and most importantly, across all models, negative self-compassion components consistently showed unique positive links to symptoms of anxiety and depression, although the strength of the edges was variable from one study to another. In contrast, of the positive self-compassion components, only self-kindness exhibited a substantial unique negative link to symptoms of depression (in Studies 1 and 3), whereas (unique) links between mindfulness/common humanity and emotional symptoms were all weak or even had been eliminated from the model. A visual inspection of the three models suggests that the nodes representing the positive self-compassion components were more or less isolated from the negative self-compassion and emotional psychopathology nodes. This was most true for the model based on the data of Study 2—in which the SCS-SF was employed, and was least the case for the model of Study 3 that relied on the SCS-Y.

Inspection of the centrality strength indicators (see Panel B of Fig. 2) showed that emotional psychopathology (Study 1: anxiety, Studies 2 and 3: depression), self-kindness (Studies 1 and Study 3), self-judgment (Study 1), overidentification (Studies 2 and 3), and isolation (Study 2) were the more central nodes, showing clear connections to other nodes included in the models. Furthermore, in all models, common humanity consistently emerged as the least central node.

## Discussion

The results of the network analyses conducted on data of three studies that each used a different variant of the SCS (i.e., original SCS, SCS-SF, and SCS-Y) generally revealed that the negative self-compassion components were more closely connected to young people’s symptoms of anxiety and depression than the positive self-compassion components. These results confirm our main hypothesis that especially the negative self-compassion components are related to psychopathology. These findings are also well in line with previous research exploring the networks of self-compassion components and emotional symptoms in adolescent samples (Barcaccia et al., 2020; Deniz et al., 2022). For example, Deniz et al., who used the SCS-Y to study (unique) links among self-compassion, depression, and well-being, also documented a network that was more or less split into two parts: one part in which self-kindness, common humanity, mindfulness, and well-being (happiness) were grouped together, and another part that consisted of self-judgment, isolation, overidentification, and depression. Altogether, network analysis provides another way of demonstrating that the negative self-compassion components have more in common with emotional psychopathology than the (protective) positive components.

Some other findings emerged from the network analysis that need some brief discussion. To begin with, in all models, the edge between anxiety and depression was particularly strong, which underlines the notion that symptoms of these emotional problems are clearly associated in adolescents (Brady & Kendall, 1992). Other results were more variable across the three studies, which could be due to sample differences, but also as a result of the variant of the SCS that was used. For instance, the model found in Study 2—in which the short form was employed—displayed the strongest split between positive self-compassion components on the one side and negative self-compassion components and emotional psychopathology on the other side. This is in agreement with Kotera and Sheffield (2020) who found evidence indicating that the SCS-SF is ‘psychometrically more fit to measure the negative aspects of self-compassion (e.g., self-criticism) than the positive aspects’ (p. 765).

Furthermore, of the two psychopathology nodes, depression appeared to be more important in the models of Studies 2 and 3, whereas in Study 1 anxiety displayed greater centrality strength. Note, however, that the latter study relied on the STAI, which has been argued not to be a pure anxiety measure as it also includes quite a number of items that assess depressive symptomatology (Bieling et al., 1998; Du Rocher & Pickering, 2022). Thus, with some caution, one might conclude that the construct of self-compassion—at least in young people—is somewhat more relevant for depression than for anxiety (Pullmer et al., 2019). However, this result may also be due to the content of the items included in the SCS (or its variants), some of which directly refer to feelings of sadness (i.e., ‘When I am feeling down …’; Neff, 2003b).

Finally, when zooming in on the positive self-compassion components, it can be concluded that self-kindness emerged as the most important variable. In two of the three studies, this node had good strength centrality (Studies 1 and 3) and also showed a quite robust negative edge with depression, suggesting a protective influence on this type of emotional psychopathology. Some scholars also view self-kindness as the core of self-compassion and try to prompt other researchers to focus more on this defining feature of the protective construct (e.g., Smith et al., 2018). In the meantime, the common humanity component seems less relevant within a context of adolescents’ emotional psychopathology: The strength centrality of this node was consistently low and no unique links with the two types of emotional problems were found. This finding might have developmental explanation (see Discussion of Part 1: common humanity has not fully emerged in adolescents), but other studies in adults have also noted that this positive self-compassion component is less strongly linked to indices of psychopathology (Muris & Petrocchi, 2017). Given that a sense of common humanity—of all self-compassion components—seems to be most firmly grounded in the Eastern culture and especially Buddhism (Segall & Kristeller, 2023), it may also be the case that this collectivistic feature is less recognizable and hence less applicable to participants in research that has mostly been conducted in individualistic Western countries.

## General Discussion

The results of the meta-analysis presented in Part 1 and the network analyses reported in Part 2 evidently reveal that the negative self-compassion components included in the SCS (and its variants) are more closely linked to emotional psychopathology in young people as compared to the positive self-compassion components. In previous papers (Muris & Otgaar, 2020, 2022), we have tried to explain why we consider this ‘differential effects’ phenomenon as problematic for the study of self-compassion as a protective construct within the context of mental health problems. Many researchers (continue to) use the scale’s total score (see also the selection process of our meta-analysis presented in Part 1: out of 57 eligible studies, only 28.1% made a distinction between positive and negative self-compassion components) and hence evince no awareness of the fact that this index in fact measures both protection *and* vulnerability. The vulnerability effects (measured by the negative self-compassion items) are simply reversed and merged with the protective influences (assessed with the positive items) and interpreted in terms of a robust positive effect of self-compassion as a shielding psychological construct.

Obviously, such a conclusion is not always valid. An illustrative example is an earlier study investigating the role of self-compassion in adolescents’ emotional problems (Muris et al., 2021). In two separate samples of non-clinical youth, it was found that the total self-compassion score (as measured with either the full-length or the short form of the SCS) was significantly and substantially negatively correlated with symptom measures of anxiety and depression (all *r*’s between -0.52 and -0.64), thus suggesting a clear-cut ‘protective effect.’ Yet, when analyzing the separate contributions of the positive and negative self-compassion components, the data convincingly showed that this observed ‘protective effect’ was driven by the reversed negative self-compassion components (i.e., vulnerability) and that the true shielding effect of the positive self-compassion components appeared to be very modest (when discarding the effect of the negative self-compassion components) or almost nonexistent (when controlling for other vulnerability and protective variables such as neuroticism, extraversion, and self-esteem). Similar findings have been documented in research studying the differential effects of positive and negative self-compassion components in adult populations suffering from depression (Körner et al., 2015), eating disorders (Bicaker & Racine, 2022), and chronic pain (Carvalho et al., 2020). Such findings again cast doubt on the protective shield that these positive self-compassion ought to have.

While acknowledging that the negative self-compassion components typically display stronger links with psychopathological problems than the positive self-compassion components, Neff (2023; see also Neff et al., 2018) continuously argues that the positive and negative self-compassion components constitute one and the same bipolar continuum (ranging from compassionate to uncompassionate self-responding), implying that it makes less sense to look at the divergent effects of positive and negative components (as more compassionate self-responding implies less uncompassionate self-responding and vice versa). She even warns that this ‘differential effects fallacy’ (Neff, 2022; p. 572) might be harmful for research because (a) the use of separate scores for positive and negative self-compassion components may complicate the findings and obscure the clear protective effect of this psychological construct, and (b) the variance explained by self-compassion will be reduced, leading to the conclusion that this variable is less relevant in the study of adaptation to adversity and stress. We have argued before that the ‘self-compassion is a bipolar construct’ argument is not valid as Neff (2003b) in her very first paper on the SCS already reported that self-kindness—self-judgment, common humanity—isolation, and mindfulness—overidentification did not emerge as dimensions (see Muris & Otgaar, 2022). This does not mean that we view the positive and negative self-compassion components as fully unrelated (as most studies do find negative associations among them), but it is also clear that they cannot be seen as fully communicating vessels, implying that an increase in self-compassionate self-responding will automatically produce an equally sized decrease in uncompassionate self-responding (and conversely). In fact, there are good theoretical arguments to consider self-compassion (soothing system) and self-criticism (threat system) as representing different (although related) emotion regulation systems (Gilbert, 2015) that are grounded in separate brain processes (Wang et al., 2022).

The warnings issued regarding the differential effects fallacy are also not very credible. While we agree with the adage that ‘there is beauty in simplicity,’ it is also well known that the etiology of psychological problems such as anxiety and depression is quite complex. For example, the currently popular developmental psychopathology account assumes that every type of abnormal behavior results from a dynamic interplay of multiple risk, vulnerability, and protective factors (Cicchetti, 2016). In our opinion, it is a strong point of the SCS (and its variants) that the measure includes components tapping vulnerability as well as protection thereby doing justice to the complexity of the origins of (emotional) psychopathology. The argument that the percentage of variance explained by self-compassion will be reduced (when discarding the negative components) and will lead to the conclusion that this variable plays a less important role is—to put it on the cautionary side—awkward. The primary purpose of psychological science is to understand the factors and processes that underlay human behavior, not about artificially boosting the variance of a variable to demonstrate its presumed importance.

A final argument to repudiate the study of differential effects of positive and negative self-compassion components is that it would have little practical value to make such a distinction (Neff, 2016, 2022, 2023). After all, self-compassion-based treatments have the primary aim to increase a person’s compassionate self-responding and—if successful—this will also decrease uncompassionate self-responding (Kirby, 2017). Indeed, the meta-analysis by Ferrari et al. (2019) indicated that there is support for this notion, although the data also pointed out that changes were quite variable across various self-compassion components (e.g., the largest effect was documented for overidentification: Hedges *g* = 0.84, while the smallest effect was noted for mindfulness: Hedges *g* = 0.40). Meanwhile, there is also evidence showing that in clinical patients with affective disorders the decrease in negative self-compassion components produced by an intervention was a better predictor of treatment outcome than the increase in positive self-compassion (Wadsworth et al., 2018). This suggests that clinicians should not only incorporate compassion-enhancing strategies in their intervention, but also deploy other therapeutic techniques that explicitly target the negative self-compassion components (e.g., cognitive restructuring, cognitive defusion) which normally are not within the scope of a self-compassion-based intervention. Thus, in clinical settings as well, it seems important to not only focus on the (promotion of) positive self-compassion but to also have an eye for the (abolishment of) negative components of self-compassion. Note also that the latter accords well with the preferences of young people who indicated that they would be more likely to engage in a treatment reducing self-criticism than in an intervention merely aiming to increase self-kindness (Egan et al., 2022).

In conclusion, the present article provides further evidence that the negative self-compassion components of the SCS (or its variants) are situated ‘on the edge of psychopathology’ and that their inclusion in the total score of this measure hinders the proper study of self-compassion as a protective construct within the context of emotional problems in young (and adult) populations. There is currently more awareness in the psychological literature that self-compassion is a quite complex construct and that the field urgently needs more sophisticated conceptualization (Cha et al., 2023; Ferrari et al., 2023a, 2023b). The separation of self-compassion in positive and negative components might be a step in the right direction and should not be considered as a ‘fallacy’ but rather as a good starting point for investigating its precise role in the etiology of psychopathological conditions in terms of both vulnerability and protection. A similar argument can be made with regard to the use of self-compassion in clinical practice: Greater awareness of the distinction between its positive and negative components may prompt clinicians to target the promotion of compassionate as well as the elimination of uncompassionate self-responding, thereby enabling them to deploy their full therapeutic arsenal to help (young) people with emotional problems.

## Data Availability

All data can be freely obtained from the first author.
